# Analysis of Cardiovascular Tissue Components for the Diagnosis of Coronary Vulnerable Plaque from Intravascular Ultrasound Images

**DOI:** 10.1155/2017/9837280

**Published:** 2017-02-08

**Authors:** Ju Hwan Lee, Yoo Na Hwang, Ga Young Kim, Eun Seok Shin, Sung Min Kim

**Affiliations:** ^1^Department of Medical Devices Industry, Dongguk University-Seoul, 26 Pil-dong 3-ga, Jung-gu, Seoul 04620, Republic of Korea; ^2^Department of Medical Biotechnology, Dongguk University-Bio Medi Campus, 32 Dongguk-ro, Ilsandong-gu, Goyang-si, Gyeonggi-do 10326, Republic of Korea; ^3^Department of Cardiology, Ulsan University Hospital, University of Ulsan College of Medicine, 877 Bangeojinsunhwando-ro, Dong-gu, Ulsan 44033, Republic of Korea

## Abstract

The purpose of this study was to characterize cardiovascular tissue components and analyze the different tissue properties for predicting coronary vulnerable plaque from intravascular ultrasound (IVUS) images. For this purpose, sequential IVUS image frames were obtained from human coronary arteries using 20 MHz catheters. The plaque regions between the intima and media-adventitial borders were manually segmented in all IVUS images. Tissue components of the plaque regions were classified into having fibrous tissue (FT), fibrofatty tissue (FFT), necrotic core (NC), or dense calcium (DC). The media area and lumen diameter were also estimated simultaneously. In addition, the external elastic membrane (EEM) was computed to predict the vulnerable plaque after the tissue characterization. The reliability of manual segmentation was validated in terms of inter- and intraobserver agreements. The quantitative results found that the FT and the media as well as the NC would be good indicators for predicting vulnerable plaques in IVUS images. In addition, the lumen was not suitable for early diagnosis of vulnerable plaque because of the low significance compared to the other vessel parameters. To predict vulnerable plaque rupture, future study should have additional experiments using various tissue components, such as the EEM, FT, NC, and media.

## 1. Introduction

Vulnerable plaques are defined as nonobstructive atherosclerotic lesions that are prone to rupture, causing acute coronary syndromes [1, 2]. Thin-cap fibroatheroma (TCFA), the hallmark of a vulnerable plaque, is characterized as a large lipid pool with an overlying thin fibrous cap (<65 *μ*m) and is heavily infiltrated by inflammatory cells and macrophages which deteriorate plaque stability [3–6].

Intravascular ultrasound (IVUS) is the gold standard for evaluating coronary plaque, lumen, and vessel characteristics [7, 8], and IVUS is an invasive imaging modality which allows the visualization of plaque morphology and collection of morphological information about the arterial wall [9]. However, although visual interpretation of grayscale IVUS can extract information on calcified tissue within plaques, and because of IVUS's inability to accurately differentiate specific plaque components, it is not easy to identify a vulnerable plaque [8, 10]. The addition of spectral analysis—virtual histology (VH)—has demonstrated a potential to provide more detailed quantitative information on plaque composition and morphology of fibrous tissue (FT), fibrofatty tissue (FFT), necrotic core (NC), and dense calcium (DC) [11]. In particular, VH-IVUS has allowed for detection of TCFA as a lesion corresponding to two criteria in at least three sequential frames: (1) focal NC lesions > 10% without evident overlying FT and (2) plaque volume ≥ 40% [12].

VH-IVUS, however, has several limitations in identifying a vulnerable plaque. First, VH-IVUS cannot visualize the TCFA due to its limited spatial resolution (>100 *μ*m). Therefore, the diagnostic accuracy for a vulnerable plaque is relatively low (≈76%) [13, 14], since it depends on the plaque characterization results, and these may not be adequate. A second limitation of VH-IVUS is from its electrocardiogram- (ECG-) gated acquisition. To minimize both radio frequency (RF) attenuation and shifting due to the presence of blood and a large amount of data, VH-IVUS depends on an ECG-gated procedure [15]. As a consequence, the RF spectrum from only one IVUS frame in each cardiac cycle is recorded which is synchronized with the R-wave in the ECG. Assuming a pullback speed of 1 mm/s and a heart rate of 60 bpm, VH acquires only one frame/s in one cardiac cycle, located at the peak R-wave [16, 17]. Therefore, the longitudinal resolution of VH-IVUS is reduced to one image out of 30 frames/s compared to the rate of the grayscale IVUS [17].

Arterial remodeling (AR) can be a good solution to the above limitations. AR provides the compensatory vessel change corresponding to plaque growth caused by positive remodeling (PR) or negative remodeling (NR) [18]. PR is usually described for an outward plaque to the edge associated with the thinning of the arterial media, whereas the NR refers to “arterial wall shrinkage” at the plaque regions [19]. Patients with acute coronary syndrome more prevalently exhibit PR and a large plaque area, while patients with stable angina more often reveal NR and a smaller plaque area [20, 21]. Moreover, ex post facto studies for coronary artery disease have validated that plaques with PR had higher lipid content and characteristics of vulnerable plaques [22, 23]. Therefore, if the relationships between the AR and various cardiovascular parameters were analyzed quantitatively, it would be possible to improve diagnostic accuracy for vulnerable plaques.

The purpose of this study was to characterize cardiovascular tissue components and analyze the different tissue properties for predicting a coronary vulnerable plaque in IVUS images. The rest of this paper is organized as follows: Section 2 introduces the details of the image acquisition, evaluation parameters, and performance validation. Sections 3 and 4 present the experimental results and discussions, respectively. Finally, Section 5 concludes the paper and identifies future works.

## 2. Materials and Methods

### 2.1. Image Acquisition

**Figure 1:**
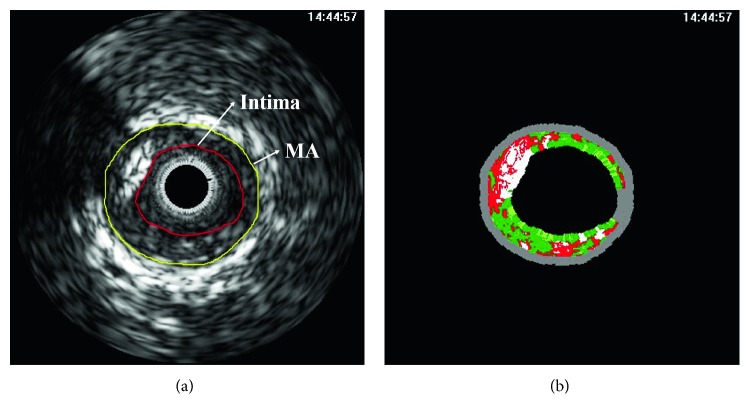
One example of the recorded IVUS images: (a) grayscale and (b) corresponding VH-IVUS images (MA: media to adventitia).

326 IVUS image frames were obtained from human coronary arteries of 14 acute coronary syndrome patients using an imaging system incorporating a 20 MHz Eagle Eye catheter (Volcano Therapeutics Inc., Rancho Cordova, CA, USA) (Figure 1). Sequential IVUS image frames were recorded along with the simultaneous ECG at 400 × 400 pixels with 8-bit grayscale. The motorized pullback speed was 0.5 mm/s, acquiring 30 frames/s. This study was approved by the Institutional Review Board (IRB) of Ulsan University Hospital, Republic of Korea.

### 2.2. Evaluation Parameters

A total of 12 evaluation parameters were estimated from the original IVUS images in order to analyze the similarities between different vessel properties (Table 1). Estimated parameters were divided into two groups including area and diameter. Lumen and vessel properties were estimated from the grayscale images, and the FT, FFT, NC, and DC parameters were obtained from the VH-IVUS images.

In addition, the external elastic membrane (EEM) was computed from all IVUS image frames to predict the vulnerable plaque after the tissue characterization. Theoretically, AR indicates dynamic changes of the EEM [24]. PR is significantly more frequent in patients with unstable coronary artery disease, whereas the NR mainly has an important role in restenosis [25]. In order to assess the extent and direction of remodeling, it is required to compare vessel size at the plaque site to an adjacent reference with minimal disease [26, 27]. PR and NR can be defined as larger or smaller EEMs at the plaque site than at a reference site. Therefore, the EEMs were estimated for each IVUS image sequence and the correlations with various vessel properties were analyzed as a preliminary study to predict the vulnerable plaque.

### 2.3. Performance Validation

**Figure 2:**
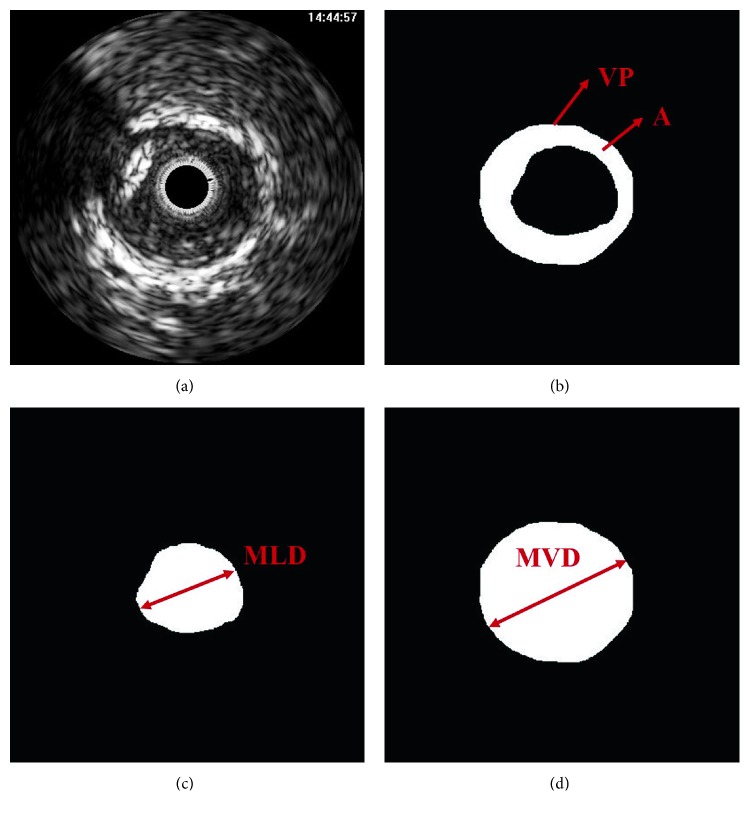
Validation indexes of the (a) original IVUS image, such as (b) area, vessel perimeter, (c) maximum lumen diameter, and (d) maximum vessel diameter.

For all IVUS images, the intima and media to adventitial (MA) borders were manually traced by two independent experts twice within a month of one another. The reliability of the manual segmentation was validated in terms of the interobserver agreement (IEA) and intraobserver agreement (IRA) of the plaque regions. IEA was estimated by analyzing the between-experts results, and the within-experts results were investigated to determine the IRA. To quantify the reliability, the validation indexes including area (A), vessel perimeter (VP), maximum lumen diameter (MLD), and maximum vessel diameter (MVD) were computed from the segmented regions (Figure 2).

### 2.4. Statistical Analyses

For manual segmentation, the IEA and IRA were analyzed using the linear regression and Bland-Altman analysis. The data were analyzed by using a paired *t*-test with the SPSS Version 21 software (SPSS Inc., Chicago, IL, USA). A *p* value of less than 0.05 was considered to be significant.

## 3. Results

### 3.1. IEA and IRA Variabilities for Manual Segmentation

Table 2 shows the IEA and IRA results of the manual tracing for all validation indexes including A, VP, MLD, and MVD for 20 MHz IVUS images. The overall average difference (AD) was greater in IEA group than that in IRA group; however, there were no significant differences. In addition, the A had the largest AD, while the MLD revealed the smallest value.

**Figure 3:**
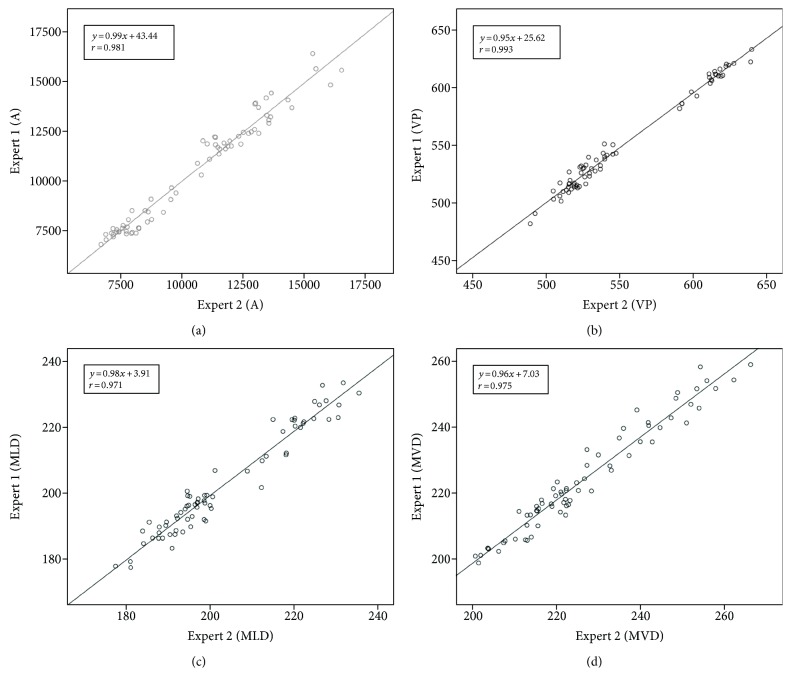
Linear regression plots for the manual segmentation between two experts (IEA) in terms of validation indexes including (a) A, (b) VP, (c) MLD, and (d) MVD.

**Figure 4:**
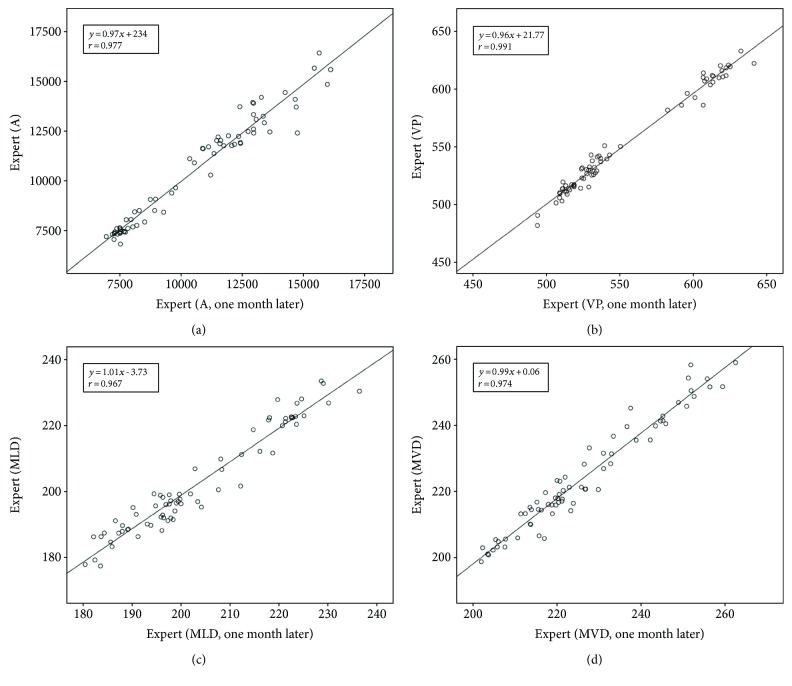
Linear regression plots for the manual segmentation within two experts (IRA) in terms of validation indexes including (a) A, (b) VP, (c) MLD, and (d) MVD.

On the other hand, all ADs were distributed within the limits of agreement (±2 SD) and were close to zero. Bland-Altman analysis indicated that the manual segmentation performed with a low mean bias and less dispersion between and within the two experts. The linear analysis also revealed that the manual segmentation had a significantly high similarity (*r* > 0.967) for all IVUS images (Figures 3 and 4). These results supported the robustness of the manual tracing for comparing the similarities between tissue properties. Figures 3 and 4 depict the linear regression plots for the IEA and IRA in terms of validation indexes, respectively.

### 3.2. Comparison of Similarities between the EEM and Evaluation Parameters

Table 3 demonstrates statistical significance levels between evaluation parameters and EEMs in terms of area and perimeter. The quantitative results revealed that the EEM was strongly correlated with the FT, NC, and media for all IVUS image sequences with a high degree of significance (*r* > 0.748), whereas the FFT and DC showed relatively low correlations (*r* < 0.586). Moreover, all lumen parameters including minimum, maximum, and average diameters had significantly low agreement compared to the EEM (*r* < 0.515). On the other hand, all vessel parameters showed significantly high correlations with the EEM area and EEM perimeter.

## 4. Discussion

Typically, VH-IVUS is regarded as the most effective method for diagnosing vulnerable plaques of acute coronary syndrome patients. Particularly, VH-IVUS can detect TCFA based on plaque compositions in at least three continuous images [12]. Although VH-IVUS is highly accurate for identifying plaque components both in vitro and in vivo, its accuracy for detecting TCFA is approximately 76% [13, 16]. In this study, we attempted to improve the diagnostic accuracy of vulnerable plaques based on the fact that AR can reveal different dynamic changes according to cardiovascular lesion types. More specifically, correlations between EEMs and 12 evaluation parameters were investigated in this study to select optimum parameters for diagnosing vulnerable plaques.

Before tissue characterization, two independent experts manually traced each border (intima and MA) twice. The first and second estimations were within a month of one another to obtain accurate plaque regions. Manual segmentation based on Bland-Altman and linear regression analyses was used to evaluate parameters including A, VP, MLD, and MVD. Variabilities between segmentation results from the two experts were very small. Their correlation coefficients were significantly high for all evaluation parameters (*r* > 0.96). In addition, Bland-Altman plots revealed that the majority of average differences were within the limits of agreement. These findings support that manual reference data are useful for comparing tissue properties with the EEM.

Results of correlation analyses between EEMs and tissue properties revealed that FT, NC, and media had statistically significant correlations with the EEM whereas FFT and DC had relatively lower correlations with the EEM for all IVUS images. Pathologic features of vulnerable plaques include positively remodeled vessel (PR) defined as a large lipid and a thin fibrous cap (<65 *μ*m) with macrophage infiltration [28, 29]. However, IVUS is unable to detect the thin fibrous cap and macrophage infiltration due to its limited spatial resolution. Therefore, it is important to find a key indicator that clearly reflects lipid characteristics for diagnosing vulnerable plaques. Regarding VH-IVUS, FFT and NC are typically regarded as lipid while FT is considered as densely packed collagen [11]. Therefore, clinicians can diagnose cardiovascular diseases based on the distribution of FFT and NC. However, our experimental results represented different aspects of plaque correlation. Although NC has been reported to be strongly correlated with PR in previous studies [30–32], FFT has relatively low correlations (*r* < 0.586) with PR. This might be due to the fact that FFT includes not only lipid components, but also collagen tissues which are not significantly correlated with PR. In other words, FFT has significant lipid interspread in collagen which may cause the abovementioned low correlation with PR. Based on these experimental results, it can be concluded that FFT is unsuitable for predicting vulnerable plaques from IVUS images.

Typically, a PR lesion has higher lipid content and macrophage count, both of which are markers of plaque vulnerability in necropsy study [22]. Results of the present study revealed that the EEM was strongly correlated with lipid content. FT and media were also found to be good indicators for diagnosing vulnerable plaques. This is a quite interesting result. As mentioned above, FT was not significantly correlated with vulnerable plaques. However, when the EEM was expanded, the volumes of plaques in lesion regions were greatly increased with increasing collagen tissues (FT) which had the largest proportion of plaque components. For this reason, the correlation tendency of FT and PR was different from that of FFT and PR due to plaque composition, not tissue property. On the other hand, lumen parameters including minimum, maximum, and average diameters presented relatively low significances in their correlations with PR. The lumen site is usually not influenced by plaque growth until the lesion reaches 40% area stenosis [24]. Lee et al. [18] have also reported low correlations between the lumen area and PR/NR at minimal luminal area site (*p* = 0.202). Therefore, the lumen does not seem to relate with AR irrespective of the worst case.

## 5. Conclusions

The quantitative results of the present study revealed that the EEM was strongly correlated with the FT, NC, and media for all IVUS image sequences with a high degree of significance, whereas the FFT and DC showed relatively low correlations. Moreover, the lumen had significantly low agreement compared to the EEM. Based on these experimental results, it was found that the FT and the media as well as NC would be a good indicator for predicting vulnerable plaque in IVUS images. In addition, the lumen was not suitable for early diagnosis of vulnerable plaque because of the low significance compared to the other vessel parameters. To predict vessel rupture, future studies should have additional experiments using various tissue components, such as AR, FT, NC, and media.

## Figures and Tables

**Table 1:** A total of 12 evaluation parameters obtained from the IVUS image sequences including area and diameter groups.

	Evaluation parameters
Area	Lumen, fibrous, fibrolipidic, lipid core, calcified, media
Diameter	Maximum lumen diameter, minimum lumen diameter, average lumen diameter, maximum vessel diameter, minimum vessel diameter, average vessel diameter

**Table 2:** IEA and IRA analyses of the manual tracing in terms of all validation indexes including A, VP, MLD, and MVD.

		A	VP	MLD	MVD
IEA	AD	43.11 ± 525.21	2.284 ± 5.68	0.91 ± 3.68	2.44 ± 3.64
	*r*	0.981	0.993	0.971	0.975
IRA	AD	38.77 ± 584.86	1.54 ± 6.11	1.02 ± 3.90	2.14 ± 3.69
	*r*	0.977	0.991	0.967	0.974

Data: mean ± standard deviation, IEA: interobserver agreement, IRA: intraobserver agreement, AD: average difference, *r*: correlation coefficient, A: area, VP: vessel perimeter, MLD: maximum lumen diameter, and MVD: maximum vessel diameter. *p* < 0.05.

**Table 3:** Comparison of the statistical significance levels between evaluation parameters and EEMs in terms of area and perimeter.

Evaluation parameters	EEM area		EEM perimeter	
	*r*	*p*	*r*	*p*
Lumen area	0.502	<0.001	0.454	<0.001
Fibrous area	0.775		0.791	
Fibrolipidic area	0.586		0.580	
Lipid core area	0.748		0.761	
Calcified area	0.520		0.534	
Media area	0.855		0.859	
Minimum lumen diameter	0.312		0.271	
Minimum vessel diameter	0.974		0.978	
Maximum lumen diameter	0.515		0.480	
Maximum vessel diameter	0.979		0.989	
Average lumen diameter	0.451		0.411	
Average vessel diameter	0.993		0.999	

*r*: correlation coefficient.
